# Predictors of human papillomavirus infection in women undergoing routine cervical cancer screening in Spain: the CLEOPATRE study

**DOI:** 10.1186/1471-2334-12-145

**Published:** 2012-06-26

**Authors:** Esther Roura, Thomas Iftner, José Antonio Vidart, Susanne Krüger Kjaer, F Xavier Bosch, Nubia Muñoz, Santiago Palacios, Maria San Martin Rodriguez, Carmen Morillo, Laurence Serradell, Laurence Torcel-Pagnon, Javier Cortes, Xavier Castellsagué

**Affiliations:** 1 Cancer Epidemiology Research Program, Institut Català d’Oncologia (ICO)-IDIBELL, CIBER-ESP, RTICC, L’Hospitalet de Llobregat, Barcelona, Catalonia, Spain; 2 Medical Virology, Section Experimental Virology, University Hospital of Tübingen, Tübingen, Germany; 3 Gynaecology Department, Hospital Clínico San Carlos, Madrid, Spain; 4 Department of Virus, Lifestyle and Genes, Danish Cancer Society Research Center, Copenhagen, Denmark; 5 Department of Gynecology, Rigshospitalet, University of Copenhagen, Copenhagen, Denmark; 6 Emeritus Professor at the National Cancer Institute, Bogotá, Colombia; 7 Instituto Palacios, Madrid, Spain; 8 Medical Department, Sanofi Pasteur MSD, Madrid, Spain; 9 Epidemiology Department, Sanofi Pasteur MSD, Lyon, France; 10 Senior Consultant in Gynaecology Oncology, Spanish Society of Obstetrics and Gynaecology, Palma de Mallorca, Islas Baleares, Spain

**Keywords:** HPV infection, Prevalence, Risk factors, Sexual behavior, Questionnaire, Spain

## Abstract

**Background:**

Human papillomavirus (HPV) is a sexually transmitted infection that may lead to development of precancerous and cancerous lesions of the cervix. The aim of the current study was to investigate socio-demographic, lifestyle, and medical factors for potential associations with cervical HPV infection in women undergoing cervical cancer screening in Spain.

**Methods:**

The CLEOPATRE Spain study enrolled 3 261 women aged 18–65 years attending cervical cancer screening across the 17 Autonomous Communities. Liquid-based cervical samples underwent cytological examination and HPV testing. HPV positivity was determined using the Hybrid Capture II assay, and HPV genotyping was conducted using the INNO-LiPA HPV Genotyping Extra assay. Multivariate logistic regression was used to identify putative risk factors for HPV infection.

**Results:**

A lifetime number of two or more sexual partners, young age (18–25 years), a history of genital warts, and unmarried status were the strongest independent risk factors for HPV infection of any type. Living in an urban community, country of birth other than Spain, low level of education, and current smoking status were also independent risk factors for HPV infection. A weak inverse association between condom use and HPV infection was observed. Unlike monogamous women, women with two or more lifetime sexual partners showed a lower risk of infection if their current partner was circumcised (P for interaction, 0.005) and a higher risk of infection if they were current smokers (P for interaction, 0.01).

**Conclusion:**

This is the first large-scale, country-wide study exploring risk factors for cervical HPV infection in Spain. The data strongly indicate that variables related to sexual behavior are the main risk factors for HPV infection. In addition, in non-monogamous women, circumcision of the partner is associated with a reduced risk and smoking with an increased risk of HPV infection.

## Background

Human papillomavirus (HPV) is one of the most common sexually transmitted infections worldwide. It is well established that HPV is the necessary cause of cervical cancer and its precancerous lesions. In addition, HPV has been associated with a wide range of other diseases including: genital warts; vulvar, vaginal, penile and anal cancers; head and neck cancers; and recurrent respiratory papillomatosis [1,2]. Most sexually active women will be infected with HPV during their lifetime [3]. While the majority of HPV infections are cleared within 2 years, persistent infections and the presence of risk factors that promote persistence may lead to the development of precancerous and cancerous lesions in a small fraction of women. HPV genotypes are classified as high-risk (HR) or low-risk (LR) based on their association with cervical cancer, HPV 16 being the type most commonly identified in this cancer [4].

Epidemiological studies conducted among women over the past decade have shown consistently that the prevalence of cervical HPV infection, particularly HR HPV types, is greatest among women below the age of 25–30 years and lower in older women [5,6]. The increased infection rates among young women are attributable to higher exposure to HPV through multiple sexual partners. In contrast, older women tend to have fewer new partners and they may have developed some degree of immunity against HPV [7]. Other potential risk factors for cervical HPV infection include tobacco smoking [8], nulliparity [9], use of oral contraceptives [9], and recent new sexual partners [10]. Use of condoms may protect against cervical HPV infection [11].

Data on cervical HPV prevalence and determinants of HPV infection are sparse and limited in Spain. In a previous analysis of the CLEOPATRE Spain study, we reported the prevalence and type-specific distribution of cervical HPV infection among women aged 18–65 years attending cervical cancer screening in Spain [12]. In the current analysis we report on the association between socio-demographic, lifestyle and medical factors and the prevalence of cervical HPV infection. Only one previous epidemiological study has been carried out in the general female population of Spain to assess these risk factors [13]. Thus, the current analysis represents the first large-scale, country-wide epidemiological study exploring risk factors for cervical HPV infection in Spain.

## Methods

### Study participants and procedures

The CLEOPATRE Spain study was a cross-sectional study conducted between June 2007 and May 2008 in 77 centers across the 17 Autonomous Communities in Spain. The study methods have been described previously in detail [12]. Briefly, women aged 18–65 years attending routine cervical cancer screening were recruited, excluding virgins and women who had a history of HPV-related cervical disease or HPV vaccination. All study participants provided written informed consent. Ethical clearance was obtained from the ethical committee of the Hospital Clínico San Carlos (Madrid, Spain).

Demographic and socio-economic data, information about sexual behavior, smoking habits, and medical history were collected after enrollment and recorded on a case report form. A liquid-based cytology (LBC) sample was collected from each participant. The LBC samples were sent to central laboratories for cytological diagnosis (Biomnis, R. Dachez, Paris, France), HPV detection (Biomnis, C. Ronsin, Ivry, France), and genotyping (Institute of Medical Virology, T. Iftner, University Hospital, Tübingen, Germany). HPV positivity was defined using the HPV DNA test, Hybrid Capture II (HC2; Digene Corporation, Gaithersburg, MD, USA). The HC2 assay uses two RNA probes, one to detect 13 HR types (16, 18, 31, 33, 35, 39, 45, 51, 52, 56, 58, 59, and 68) and another one to detect 5 LR types (6, 11, 42, 43, and 44) in cervical samples [14]. Samples were considered to be HPV-positive if either of the two probes (low- and/or high-risk probes) were positive at a relative light unit coefficient (RLU/Co) of ≥1.0, which corresponds to the HPV DNA threshold of 1.0 pg/mL recommended by the United States Food and Drug Administration (US FDA). HPV genotyping was determined in all study samples using the INNO-LiPA HPV Genotyping Extra assay (Innogenetics, Gent, Belgium). Only results of HPV genotyping for the HPV-HC2-positive women are shown. The INNO-LiPA test allows identification of 13 established HR HPV types (16, 18, 31, 33, 35, 39, 45, 51, 52, 56, 58, 59, and 66), 5 known or putative HR types (26, 53, 68, 73, and 82), 7 LR HPV types (6, 11, 40, 43, 44, 54, and 70), additional non-differentiated HPV types, and types with undefined risk (74 and 69/71). Automated results were confirmed with blinded manual readings undertaken by experienced laboratory personnel.

### Risk factor analysis

The evaluation of potential risk factors included age, type of community (rural, suburban or urban based on population size of municipality), country of birth, marital status, level of education, professional status, smoking status (including duration and intensity), pregnancies, contraceptive methods, lifetime number of sexual partners, age at first sexual intercourse, time since first sexual intercourse, history of cervical lesions, history of sexually transmitted infections (STIs), current partner’s history of STIs in the last 12 months, current partner’s circumcision status, and immune status.

The association between cervical HPV infection and potential risk factors was tested by multivariate analysis, which involved construction of an adjusted logistic regression model using stepwise regression with forward selection. Beginning with a basic model adjusted for age and Autonomous Community, the variables for the potential risk factors were added one at a time into the model to evaluate their association with cervical HPV infection, and those that showed a statistically significant association (P-value <0.10) were retained in the final model as adjusting variables.

Adjusted models were also constructed to evaluate the association between potential risk factors and HR HPV, LR HPV, HPV 16, and single and multiple HPV infections. Analyses were conducted to explore multiplicative interactions between lifetime number of sexual partners and other potential risk factors for cervical HPV infection. In addition, an adjusted model was constructed to assess risk factors for HPV positivity among young women aged 18–25 years.

Analyses were performed using the R programing language (R Development Core Team, 2005, http://www.r-project.org).

## Results

### Study participants

A total of 7 252 women were initially registered in the recruitment logs at the participating sites. Of those, 4 206 were eligible and 3 261 were finally enrolled in the study. The participation rate was 77.5% (3 261/4 206). Reasons for non-participation among eligible women were: refusal (13.2%), and other reasons such as logistic problems or menstruation (9.3%). The characteristics of study participants have been described previously [12]. In brief, 91.3% of the women were born in Spain, 77.2% lived in an urban area (≥10 000 inhabitants), 39.7% were single, 33.0% had a university/college education, 25.5% were students, 35.6% were current smokers, and 19.5% reported having had four or more lifetime sexual partners. There was a planned oversampling of young women aged ≤25 years (49.6%) compared with the proportion of this age group in the overall female population of Spain (18.0%).

### Prevalence of HPV

HC2 results were available for 3 155 (96.7%) women [12]. A total of 608 (19.3%) women were HPV-positive based on the HC2 assay, 527 of whom (86.7%) were infected with one or more HR types (with or without concomitant LR HPV infection). LiPA results were available for 606 HC2-positive samples (497 LiPA positive and 109 LiPA negative). Single and multiple HPV infections were detected in 49.2% (298/606) and 32.8% (199/606) of these samples, respectively.

### Risk factor analysis

#### Risk factors for any HPV infection

In the basic model adjusted for age and Autonomous Community, the following characteristics were positively and statistically significantly associated with cervical HPV infection: young age; living in an urban community; country of birth other than Spain; unmarried status; current smoker; more than one lifetime sexual partner; young age at first sexual intercourse (with a statistically significant trend); more years elapsed since first intercourse (with a statistically significant trend); and a history of cervical lesions, genital warts or other STIs (Table 1). There was also a weak and statistically borderline inverse association between systematic use of condoms in the last 12 months, meaning use of condom in 90-100% of all sexual relationships that occurred in the last year, and cervical HPV infection. No associations were identified between cervical HPV infection and number of pregnancies, use of oral contraceptives, ever use of condoms or a current sexual partner who was circumcised or with STIs in the last 12 months. Other variables, including type of medical practice and setting, professional status, duration and intensity of smoking, use of an intrauterine device, and immune status, were also assessed but no associations with HPV infection were observed (data not shown).

**Table 1:** Prevalence of cervical human papillomavirus infection and crude associations between infection and selected subjects’ characteristics

**Variables**	**No. of HPV- positive samples/ no. of tested samples**	**HPV prevalence, %**	**Basic model**^**1**^	
			**POR**	**95% CI**
Age group (years)				
18	80/308	26.0	**4.8**	**3.1–7.5**
19	101/339	29.8	**5.7**	**3.7–8.8**
20–21	131/452	29.0	**5.5**	**3.6–8.3**
22–25	137/471	29.1	**5.5**	**3.6–8.2**
26–35	54/319	16.9	**2.7**	**1.7–4.3**
36–45	31/326	9.5	1.4	0.8–2.3
46–55	42/486	8.6	1.2	0.8–2.0
56–65	32/454	7.0	1	reference
*P-value for trend*			***<0.001***	
Residential area				
Rural	48/329	14.6	1	reference
Suburban	71/393	18.1	1.3	0.8–1.9
Urban	488/2428	20.1	**1.6**	**1.1–2.2**
Unknown	5	-	-	-
*P-value for trend*			***0.005***	
Country of birth				
Spain	525/2886	18.2	1	reference
Other countries	83/269	30.9	**1.7**	**1.3–2.3**
Marital status				
Single	361/1259	28.7	**3.4**	**2.4–4.9**
Cohabiting	137/549	25.0	**2.9**	**2.0–4.3**
Married	71/1158	6.1	1	reference
Divorced/Separated	28/127	22.0	**4.4**	**2.7–7.1**
Widowed	11/62	17.7	**3.9**	**1.9–8.0**
Level of educational attainment				
No formal education	27/178	15.2	1	reference
Primary completed	92/648	14.2	0.9	0.5–1.4
Secondary completed	290/1286	22.6	1.0	0.6–1.5
University or higher	199/1040	19.1	0.7	0.5–1.2
Unknown	3	-	-	-
*P-value for trend*			*0.07*	
Tobacco smoking status				
Never smoked	227/1599	14.2	1	reference
Ex-smoker	57/439	13.0	1.2	0.8–1.6
Current smoker	324/1116	29.0	**2.3**	**1.8–2.8**
Unknown	1	-	-	-
*P-value for trend*			***<0.001***	
Number of pregnancies				
0	330/1340	24.6	1	reference
1	93/417	22.3	**1.3**	**1.0–1.8**
2	62/587	10.6	1.0	0.7–1.4
≥3	43/511	8.4	0.8	0.5–1.3
Unknown	300	-	-	-
*P-value for trend among parous women*			*0.3*	
Use of oral contraceptives				
Never used	240/1299	18.5	1	reference
Ex-user	192/1141	16.8	1.2	1.0–1.5
Current user	176/712	24.7	1.0	0.8–1.2
Unknown	3	-	-	-
Ever use of condoms				
Never used	85/650	13.1	1	reference
Ex-user	162/1003	16.2	1.0	0.7–1.4
Current user	361/1502	24.0	1.1	0.8–1.5
Systematic condom use in last 12 months^*^				
No	359/1947	18.4	1	reference
Yes	226/1031	21.9	**0.8**	**0.6–1.0**
Unknown	12	-	-	-
Lifetime number of sexual partners				
1	139/1560	8.9	1	reference
2	103/577	17.9	**1.8**	**1.4–2.4**
3	122/397	30.7	**3.9**	**2.9–5.3**
≥4	241/609	39.6	**5.8**	**4.5–7.5**
Unknown	12	-	-	-
*P-value for trend*			***<0.001***	
Age at first sexual intercourse (years)				
≤14	41/125	32.8	**2.1**	**1.0–4.5**
15–16	192/685	28.0	1.6	0.8–3.1
17–18	235/1097	21.4	1.3	0.7–2.5
19–20	69/492	14.0	1.2	0.6–2.3
21–25	58/595	9.7	1.1	0.6–2.2
≥26	11/145	7.6	1	reference
Unknown	16	-	-	-
*P-value for trend*			***0.006***	
Current partner had STIs in last 12 months				
No	538/2888	18.6	1	reference
Yes	9/38	23.7	1.1	0.5–2.4
Unknown	229	-	-	-
Current partner circumcised				
No	432/2366	18.3	1	reference
Yes	60/369	16.3	0.9	0.6–1.2
Unknown	420	-	-	-
Previous cervical lesions				
No	559/2952	18.9	1	reference
Yes	36/178	20.2	**1.7**	**1.1–2.5**
Unknown	25	-	-	-
History of genital warts				
No	555/3061	18.1	1	reference
Yes	50/87	57.5	**4.8**	**3.1–7.6**
Unknown	7	-	-	-
History of other STIs				
No	579/3054	19.0	1	reference
Yes	23/87	26.4	**1.7**	**1.0–2.8**
Unknown	14	-	-	-

HPV, human papillomavirus; POR, prevalence odds ratio; CI, confidence interval; STI, sexually transmitted infection.

^1^Basic model: adjusted for age and Autonomous Community.

^*^Among women with a sexual partner in the last 12 months.

Bold font indicates a significant effect (*P* < 0.05).

In the final adjusted model, variables that remained statistically significant were: age 18–25 years; living in an urban community; country of birth other than Spain; unmarried status; lower level of educational attainment (with a statistically significant trend); current smoker; more than one lifetime sexual partner; and a history of genital warts (Table 2). A high lifetime number of sexual partners, a history of genital warts, and unmarried status were the risk factors most strongly associated with cervical HPV infection. Notably, women who had four or more sexual partners had a 4-fold higher risk of being HPV-positive than women who had only one sexual partner.

**Table 2:** Multivariate analyses of the association between cervical human papillomavirus infection and selected subjects’ characteristics

	**No. of tested samples**	**Adjusted model**^1^	
		**POR**	**95% CI**
Age group (years)			
18	308	**1.7**	**1.1–3.1**
19	339	**2.2**	**1.4–3.7**
20–21	452	**2.1**	**1.3–3.4**
22–25	471	**1.9**	**1.2–3.2**
26–35	319	1.1	0.7–1.9
26–45	326	0.7	0.4–1.2
46–55	486	0.8	0.5–1.3
56–65	454	1	reference
*P-value for trend*		***<0.001***	
Residential area			
Rural	329	1	reference
Suburban	393	1.3	0.8–2.1
Urban	2428	**1.5**	**1.0–2.2**
Unknown	5	-	-
*P-value for trend*		***0.03***	
Country of birth			
Spain	2886	1	reference
Other countries	269	**1.7**	**1.3–2.4**
Marital status			
Married	1158	1	reference
Single	1259	**2.1**	**1.4–3.1**
Cohabiting	549	**1.8**	**1.2–2.6**
Divorced/Separated	127	**2.3**	**1.4–3.4**
Widowed	62	**3.7**	**1.7–7.7**
Level of education attainment			
No formal education	178	1	reference
Primary completed	648	0.9	0.5–1.5
Secondary completed	1286	0.9	0.5–1.4
University or higher	1040	0.7	0.4–1.1
Unknown	3	-	-
*P-value for trend*		***0.02***	
Tobacco smoking status			
Never smoked	1599	1	reference
Ex-smoker	439	1.0	0.7–1.3
Current smoker	1116	**1.6**	**1.3–1.9**
Unknown	1	**-**	**-**
*P-value for trend*		***<0.001***	
Ever use of condoms			
Never used	650	1	reference
Ex or current user	2505	0.9	0.6–1.1
Systematic condom use in last 12 months^*^			
No	1947	1	reference
Yes	1031	**0.8**	**0.6–1.0**
Unknown	12	-	-
Lifetime number of sexual partners			
1	1560	1	reference
2	577	**1.5**	**1.1–2.1**
3	397	**3.3**	**2.5–4.5**
≥4	609	**4.1**	**3.1–5.4**
Unknown	12	-	-
*P-value for trend*		***<0.001***	
Age at first sexual intercourse (years)			
≤14	125	0.5	0.2–1.2
15–16	685	0.6	0.3–1.2
17–18	1097	0.7	0.3–1.4
19–20	492	0.8	0.4–1.6
21–25	595	1.0	0.5–2.1
≥26	145	1	reference
Unknown	16	-	-
*P-value for trend*		***0.003***	
Current partner circumcised			
No	2366	1	reference
Yes	369	0.8	0.6–1.1
Unknown	420	-	-
History of genital warts			
No	3061	1	reference
Yes	87	**3.2**	**2.0–5.2**
Unknown	7	-	-

POR, prevalence odds ratio; CI, confidence interval.

^1^Adjusted model: adjusted for age, Autonomous Community, country of birth, marital status, level of education, smoking habits, lifetime number of sexual partners, and history of genital warts.

^*^Among women with a sexual partner in the last 12 months.

Bold font indicates a significant effect (*P* < 0.05).

**Figure 1:**
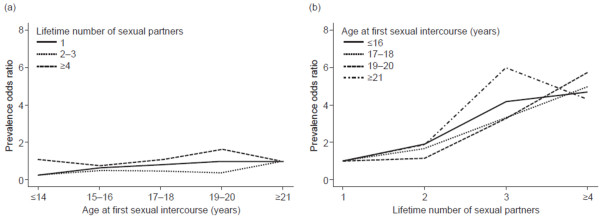
Prevalence odds ratios for human papillomavirus*. (a) The association between cervical HPV infection and age at first sexual intercourse stratified by lifetime number of sexual partners. Reference group: ≥21 years of age at first sexual intercourse. **(b)** The association between cervical HPV infection and lifetime number of sexual partners stratified by age at first sexual intercourse. Reference group: 1 sexual partner. *P* < 0.001 for association for each age stratum. *Model adjusted for age, Autonomous Community, country of birth, marital status, level of education, smoking habits, and history of genital warts.

It is interesting to note that the pattern of association with age at first intercourse in the adjusted model was opposite to that found in the basic model. While in the crude analysis young age at first intercourse was associated with increased HPV prevalence, in the fully adjusted model this effect was reversed and young age at first intercourse was associated with reduced HPV prevalence (P for trend = 0.003) (Table 2). To explore these associations further, we performed stratified analyses by levels of lifetime number of sexual partners and levels of age at first sexual intercourse (Figure 1). As shown in Figure 1a, HPV prevalence was not associated with age at first intercourse within each category of number of sexual partners. In contrast, Figure 1b shows that HPV prevalence was strongly associated with lifetime number of sexual partners within each category of age at first intercourse (*P* < 0.001 for each age stratum). These stratified analyses show that HPV prevalence is more strongly associated with lifetime number of sexual partners than with age at first sexual intercourse. A similar effect was obtained when evaluating time since first sexual intercourse instead of age at first intercourse.

#### Interaction between lifetime number of sexual partners, male circumcision, and smoking status

Interaction analyses showed that having a current partner who was circumcised had a significant protective effect against cervical HPV infection in women reporting two or more lifetime sexual partners but not in monogamous women (*P* for interaction = 0.005) (Table 3). A synergistic interaction with cervical HPV infection was also observed between smoking and having two or more lifetime sexual partners. Thus, the risk of infection almost doubled in women reporting two or more lifetime sexual partners if they were current smokers, whereas in monogamous women current smokers showed little increase in risk compared with non-smokers (*P* for interaction = 0.01). No other statistically significant interactions with lifetime number of sexual partners were found.

**Table 3:** Association between male circumcision, smoking, and cervical human papillomavirus infection by number of sexual partners

	**HPV infection POR (95% CI)**		*P*-value of interaction*
	**1 sexual partner**	**≥2 sexual partners**	
Current partner circumcised			0.005
No (reference)	1.0	1.0	
Yes	1.6 (1.0–2.7)	**0.6 (0.4–0.9)**	
Tobacco smoking status			0.01
Never/ex-smoker (reference)	1.0	1.0	
Current smoker	1.1 (0.7–1.7)	**2.0 (1.6–2.5)**	

POR, prevalence odds ratio; CI, confidence interval.

*Log-Likelihood test.

Model adjusted for age, Autonomous Community, country of birth, marital status, level of education, smoking habits, lifetime number of sexual partners, and history of genital warts.

Bold font indicates a significant effect (P<0.05).

#### Risk factors for HPV infection in young women

We examined the potential risk factors associated with HPV infection among young women aged ≤25 years. In the adjusted model, the lifetime number of sexual partners, a history of genital warts, a country of birth other than Spain, and current smoking status were identified as the most important risk factors for cervical HPV infection (data not shown). Young age at first intercourse was associated with decreased HPV prevalence (*P* for trend = 0.006). Accordingly, increasing time since first sexual intercourse was also inversely associated with HPV infection (*P* for trend = 0.06).

#### Risk factors for HR versus LR HPV infections and for HPV 16 infections

We explored whether the determinants for HR HPV infection differed from those for LR infection by carrying out a stratified analysis (Table 4). The risk factors for HR infections (Table 4) were the same as those for any HPV infection (Table 2). In contrast, the determinants for LR HPV infection were: history of genital warts, increasing lifetime number of sexual partners, being single, and (inversely but weakly) ever use of condoms (Table 4). We also compared the risk factors for HR versus LR HPV infections by modeling infected women only. The following factors were positively associated with HR as compared to LR infection: birth in countries other than Spain, ever use of condoms, and a history of previous cervical lesions (data not shown)*.*

**Table 4:** Multivariate analyses of the association between high- and low-risk human papillomavirus infections and selected subjects’ characteristics

	**Any HR HPV vs. negative (n = 3074)**			**Only LR HPV vs. negative (n = 2628)**		
	**No. tested**	**Adjusted model**^**1**^		**No. tested**	**Adjusted model**^**1**^	
		**POR**	**95% CI**		**POR**	**95% CI**
Age group (years)						
18	302	**2.3**	**1.3–4.1**	234	0.7	0.2–2.3
19	327	**2.7**	**1.5–4.7**	250	1.4	0.5–4.0
20–21	440	**2.6**	**1.5–4.5**	333	0.9	0.3–2.6
22–25	449	**2.2**	**1.3–3.8**	356	1.6	0.6–4.2
26–35	312	1.3	0.7–2.3	272	0.7	0.2–2.3
36–45	325	0.9	0.5–1.7	296	0.1	0.0–0.8
46–55	474	0.8	0.4–1.4	456	1.1	0.4–2.8
56–65	445	1	reference	431	1	reference
*P-value for trend*		***<0.001***			*0.8*	
Residential area						
Rural	324	1	reference	286	1	reference
Suburban	383	1.3	0.8–2.1	332	1.5	0.5–4.7
Urban	2362	**1.5**	**1.0–2.2**	2006	1.5	0.6–4.1
Unknown	5	-	-	4	-	-
*P-value for trend*		***0.03***			*0.4*	
Country of birth						
Spain	2811	1	reference	2436	1	reference
Other countries	263	**2.0**	**1.4–2.8**	192	0.6	0.2–1.6
Marital status						
Married	1143	1	reference	1102	1	reference
Single	1214	**2.1**	**1.4–3.2**	943	**2.8**	**1.1–7.1**
Cohabiting	533	**1.7**	**1.1–2.7**	428	2.3	0.9–5.9
Divorced/ Separated	125	**2.7**	**1.5–4.7**	101	0.8	0.2–3.6
Widowed	59	**4.0**	**1.7–9.3**	54	3.4	0.9–13.1
Level of educational attainment						
No formal education	177	1	reference	152	1	reference
Primary completed	640	0.9	0.5–1.5	564	2.2	0.3–18.5
Secondary completed	1241	0.7	0.4–1.2	1041	5.6	0.7–42.4
University or higher	1013	**0.6**	**0.3–1.0**	868	3.8	0.5–30.1
Unknown	3	-	-	3	-	-
*P-value for trend*		***0.003***			*0.2*	
Tobacco smoking status						
Never smoked	1566	1	reference	1405	1	reference
Ex-smoker	426	0.9	0.6–1.3	395	1.4	0.7–2.8
Current smoker	1081	**1.6**	**1.3–2.1**	827	1.2	0.7–2.0
Unknown	1	-	-	1	-	-
*P-value for trend*		***<0.001***			*0.5*	
Ever use of condoms						
Never used	630	1	reference	585	1	reference
Ex or current user	2444	0.9	0.7–1.3	2043	**0.5**	**0.3–1.0**
Systematic condom use in last 12 months^*^						
No	1902	1	reference	1633	1	reference
Yes	1001	**0.8**	**0.6–1.0**	835	1.0	0.6–1.7
Unknown	11	-	-	12	-	-
Lifetime number of sexual partners						
1	1534	1	reference	1447	1	reference
2	564	**1.6**	**1.2–2.2**	487	1.2	0.6–2.4
3	383	**3.5**	**2.6–4.9**	289	**2.6**	**1.3–5.2**
≥4	581	**4.4**	**3.3–5.9**	396	**3.0**	**1.6–5.6**
Unknown	12	-	-	9	-	-
*P-value for trend*		***<0.001***			***<0.001***	
Age at first sexual intercourse (years)						
≤14	121	0.7	0.3–1.8	88	0.2	0.0–1.2
15–16	671	0.8	0.3–1.9	507	**0.2**	**0.1–0.8**
17–18	1061	0.9	0.4–2.0	898	0.5	0.1–1.6
19–20	481	1.0	0.4–2.5	434	0.5	0.1–1.8
21–25	584	1.3	0.6–3.2	548	0.6	0.2–1.9
≥26	141	1	reference	138	1	reference
Unknown	15	-	-	15	-	-
*P-value for trend*		***0.02***			***0.01***	
Current partner circumcised						
No	2311	1	reference	1989	1	reference
Yes	359	0.7	0.5–1.0	319	1.2	0.6–2.4
Unknown	404	-	-	320	-	-
History of genital warts						
No	2991	1	reference	2576	1	reference
Yes	78	**2.9**	**1.7–4.9**	46	**6.6**	**2.8–15.4**
Unknown	5	-	-	6	-	-

HPV, human papillomavirus; HR, high-risk; LR, low-risk; POR, prevalence odds ratio; CI, confidence interval.

^1^Adjusted model: adjusted for age, Autonomous Community, country of birth, marital status, level of education, smoking habits, lifetime number of sexual partners, and history of genital warts.

^*^Among women with a sexual partner in the last 12 months

Bold font indicates a significant effect (*P* < 0.05).

Determinants associated with HPV 16 infection, compared with HPV-negative women, were also evaluated. Women of young age, smokers, and women with more than two lifetime sexual partners had an increased risk of HPV 16 infection (data not shown).

#### Risk factors for single versus multiple HPV infections

In a stratified adjusted model, the same variables as those identified in the adjusted model for any HPV infection (Table 2) were independently associated with single or multiple HPV infections (Table 5), except for residential area. Furthermore, ever use of condoms had a borderline protective effect against single HPV infection but not against multiple infection. Regression models comparing potential risk factors for multiple versus single HPV infections showed that women with more than two lifetime sexual partners were more likely to have multiple than single HPV infections (data not shown).

**Table 5:** Multivariate analyses of the association between single and multiple human papillomavirus infections and selected subjects’ characteristics

	**Single infection vs. negative (n = 2845)**			**Multiple infection vs. negative (n = 2746)**		
	**No. tested**	**Adjusted model**^1^		**No. tested**	**Adjusted model**^1^	
		**POR**	**95% CI**		**POR**	**95% CI**
Age group (years)						
18	268	**3.1**	**1.4–6.8**	257	**3.7**	**1.2–11.8**
19	287	**3.5**	**1.6–7.5**	273	**4.1**	**1.3–12.9**
20–21	382	**3.1**	**1.5–6.5**	369	**4.0**	**1.3–12.5**
22–25	401	**3.1**	**1.5–6.4**	387	**3.8**	**1.2–11.7**
26–35	301	**2.4**	**1.1–4.9**	279	1.5	0.4–4.9
36–45	309	0.9	0.4–2.1	304	1.4	0.4–5.0
46–55	464	1.1	0.5–2.5	451	0.97	0.3–3.5
56–65	433	1	reference	426	1	reference
*P-value for trend*		***<0.001***			***0.001***	
Residential area						
Rural	305	1	reference	301	1	reference
Suburban	359	1.3	0.7–2.4	344	0.9	0.4–2.0
Urban	2176	1.4	0.9–2.4	2097	1.0	0.6–1.9
Unknown	5	-	-	4	-	-
*P-value for trend*		*0.2*			*0.8*	
Country of birth						
Spain	2616	1	reference	2528	1	reference
Other countries	229	**2.0**	**1.3–3.0**	218	**2.2**	**1.3–3.5**
Marital status						
Married	1120	1	reference	1097	1	reference
Single	1073	**2.4**	**1.5–4.1**	1034	**3.2**	**1.4–7.2**
Cohabiting	483	**2.1**	**1.3–3.6**	452	2.0	0.9–4.7
Divorced/Separated	114	**2.6**	**1.3–5.2**	110	**5.4**	**2.1–14.2**
Widowed	55	**3.5**	**1.1–11.0**	53	**7.0**	**1.3–37.9**
Level of education attainment						
No formal education	160	1	reference	162	1	reference
Primary completed	600	1.3	0.6–3.0	584	0.7	0.3–1.5
Secondary completed	1139	1.3	0.6–2.8	1095	0.6	0.3–1.4
University or higher	943	1.1	0.5–2.3	902	**0.5**	**0.2–1.0**
Unknown	3	-	-	3	-	-
*P-value for trend*		*0.4*			***0.03***	
Tobacco smoking status						
Never smoked	1478	1	reference	1438	1	reference
Ex-smoker	412	1.1	0.7–1.7	398	1.0	0.5–1.8
Current smoker	954	**1.7**	**1.3–2.3**	909	**1.6**	**1.1–2.4**
Unknown	1	-	-	1	-	-
*P-value for trend*		***<0.001***			***0.005***	
Ever use of condoms						
Never used	612	1	reference	582	1	reference
Ex or current user	2233	**0.7**	**0.4–1.0**	2164	1.1	0.6–1.9
Systematic condom use in last 12 months^*^						
No	1761	1	reference	1707	1	reference
Yes	921	0.8	0.6–1.1	880	0.7	0.5–1.0
Unknown	12	-	-	11	-	-
Lifetime number of sexual partners						
1	1487	1	reference	1447	1	reference
2	528	**1.6**	**1.1–2.4**	504	**2.3**	**1.3–3.9**
3	337	**3.4**	**2.3–5.0**	319	**6.0**	**3.5–10.2**
≥4	482	**3.7**	**2.6–5.4**	466	**8.1**	**5.0–13.3**
Unknown	11	-	-	10	-	-
*p-value for trend*		***<0.001***			***<0.001***	
Age at first sexual intercourse (years)						
≤14	104	0.7	0.2–2.3	100	0.4	0.1–2.4
15–16	589	0.8	0.3–2.5	567	0.6	0.1–2.8
17–18	973	0.9	0.3–2.6	941	0.7	0.1–3.3
19–20	459	1.1	0.3–3.2	440	0.8	0.2–3.7
21–25	567	1.4	0.5–4.2	548	1.0	0.2–4.8
≥26	138	1	reference	136	1	reference
Unknown	15	-	-	14	-	-
*P-value for trend*		***0.03***			*0.07*	
Current partner circumcised						
No	2138	1	reference	2079	1	reference
Yes	343	0.9	0.6–1.4	327	0.7	0.4–1.3
Unknown	364	-	-	340	-	-
History of genital warts						
No	2780	1	reference	2684	1	reference
Yes	59	**2.8**	**1.5–5.1**	58	**4.4**	**2.3–8.8**
Unknown	6	-	-	4	-	-

POR, prevalence odds ratio; CI, confidence interval.

^1^Adjusted model: adjusted for age, Autonomous Community, country of birth, marital status, level of education, smoking habits, lifetime number of sexual partners, and history of genital warts.

^*^Among women with a sexual partner in the last 12 months.

Bold font indicates a significant effect (*P* < 0.05).

## Discussion

This is the first large-scale, country-wide study in Spain to evaluate potential risk factors for cervical HPV infection. In addition to young age, a lifetime number of two or more sexual partners, a history of genital warts, and being unmarried were the strongest risk factors for cervical HPV infection among women aged 18–65 years attending routine cervical cancer screening across Spain. Living in an urban community, country of birth other than Spain, low level of education, and current smoking were also identified as independent risk factors for cervical HPV infection. Condom use provided a borderline protective effect against HPV infection.

Our results agree with those of a previous epidemiological study in the general female population of Spain (N = 973) conducted in 2003 by de Sanjose et al., which found the following independent risk factors of HPV infection: overseas birth, divorce, more than one sexual partner, and smoking marijuana or related products. Use of condoms with a regular partner was found to be protective against HPV, but with a borderline effect [13].

As in other studies [7,10,11,13], an increased lifetime number of sexual partners was the most consistent risk factor for cervical HPV infection identified in our study. In contrast, age at first sexual intercourse was not identified as an independent risk factor in the multivariate analysis, in accordance with some previous studies [7,10,13]. In our study, young age at first intercourse increased the risk of HPV infection in the unadjusted model but in the adjusted one the effect was reversed. The inclusion of number of sexual partners in the adjusted model explained this change, indicating reverse co-linearity between the two variables. Our further stratified analyses, as shown in Figure 1, strongly suggested that the key determinant of cervical HPV infection is the number of sexual partners the woman had had and not the age at first sexual intercourse. These findings are in agreement with other studies suggesting that age at sexual debut is a likely predictor of lifetime number of sexual partners and not a factor independently associated with cervical HPV infection [15,16]. A similar effect was observed when time since first sexual intercourse was assessed rather than age at first sexual intercourse.

Country of birth other than Spain was identified as an independent risk factor for cervical HPV infection, a finding that was reported in a previous, smaller study conducted in Spain [13]. In our study, most women born outside Spain were immigrants from Latin America. The HPV prevalence in these countries is higher than that in Spain [17,18]. The increased prevalence of HPV among immigrant women compared with women born in Spain may be related to well-known differences in sexual behavior in both men and women.

In agreement with the pooled analysis of the International Agency for Research on Cancer (IARC) on smoking and HPV prevalence [8], we identified current smoking status as an independent risk factor for cervical HPV infection. A large population-based study conducted in women from Costa Rica found a positive association between smoking status and HPV 16, an association that was also found to be statistically significant in our study [10]. Intensity of smoking was identified as another predictor of HPV infection in the IARC analysis, but these data were not statistically significant in our adjusted model. An association between smoking and cervical HPV infection is difficult to assess because of the strong confounding effect of sexual behavior. To address this we performed further logistic regression analyses to formally test for the interaction between smoking and number of sexual partners, and found a statistically significant interaction between the two risk factors (Table 3). Smoking was associated with a two-fold increased prevalence of cervical HPV in women with multiple sexual partners but not among monogamous women. In contrast, in the IARC study a significant relationship of smoking intensity was seen only among women who reported one sexual partner, but not among women reporting more partners. A possible explanation for this interaction is that among women with a high exposure to HPV (those with multiple partners) the risk of infection is much increased if they are concomitantly exposed to smoking. Conversely, smoking in the absence of high exposure to HPV is not as relevant in determining the risk of HPV infection. Some studies have documented that in the presence of HPV, tobacco products may reduce local immunological responses, which might facilitate HPV acquisition, reduce HPV clearance, or increase HPV persistence, thus increasing the overall HPV prevalence [19,20].

Condom use was found to be inversely but weakly associated with cervical HPV infection. The protective effect of condom use against HPV infection is controversial in the literature. Whereas some previous studies have reported a weak association with HPV infection [10,11,13], others have not [7]. Taking into account the weak magnitude of the associations found overall and in the subgroup analyses performed in our study, the beneficial effect of condom use on HPV prevalence is probably small.

Although male circumcision was not identified as a predictor of cervical HPV infection in our study, interaction analyses showed a protective effect among women at increased risk of HPV infection (i.e. women who had more sexual partners). Previous studies have shown that circumcised men are less likely to have penile HPV infection than uncircumcised men and that male circumcision infers a protective effect in female sexual partners for cervical HPV infections as well as for cervical cancer [21,22].

A strength of this study is that the age-stratified sampling strategy used within each of the Autonomous Communities of Spain (except Ceuta and Melilla) provided a good demographic representation of the age-specific female population attending cervical cancer screening across the country. However, a potential limitation is that women were selected from opportunistic cervical cancer screening programs, which may have resulted in the preferential recruitment of health-conscious or healthier women. This potential over-representation of “healthy” women could underestimate the true prevalence of risk factors related to cervical HPV infection, such as tobacco smoking, high number of sexual partners or having never used a condom. The potential effect of this would be an underestimation of the true effects related to these risk factors. In order to assess potential selection bias, the distribution of key socio-demographic and lifestyle characteristics of women included in the CLEOPATRE study was compared with that of the general female population of Spain using public data such as marital status, parity, use of contraceptive methods, and number of sexual partners among others. We ascertained that the study population was indeed representative of the general female population in Spain (data not shown). As with any other cross-sectional design, there is a risk of reporting bias, particularly in relation to variables that address aspects of sexual behavior, such as “age at first sexual intercourse,” “lifetime number of sexual partners,” and “partner’s circumcision status.” Other studies have noted also the possibility of this non-differential misclassification related to sexual behavior [7]. It is well known that non-differential misclassification cannot inflate true effects; instead, it would shift the observed association towards the null value.

Another limitation of our study is that only 80% of the HC2-positive samples were positive by LiPA, a much more sensitive assay than HC2. This apparently low performance of the LiPA assay may be explained by the inherent differences between the two assays. Although the HC2 assay is designed to detect 18 HPV types (including the low-risk types), several studies have noted that the HC2 HR probe mix may cross-react with other HPV types that are not represented in the mix [23-25]. Data on the cross-reactivity of the low-risk probe are not available, but it can be assumed that cross-reactivity also takes place with that probe. In contrast, in the LiPA test (which is designed to detect 27 HPV genotypes by identification of highly specific HPV DNA sequences), cross-reactivity with other HPV types has not been observed. It is thus conceivable that HPV types detected by HC2 through cross-hybridization are not detectable with the LiPA assay. The HR probe and the LR probe, with an unknown extent of cross-reactivity, were both used in the present study, and this may explain the observed relatively low sensitivity of the LiPA assay. When comparing the two methods, a Kappa value of 0.67 (95% CI, 0.64–0.70) was found, which could be interpreted as being acceptable.

## Conclusion

This analysis of the CLEOPATRE Spain study has identified several socio-demographic, lifestyle, and medical factors associated with cervical HPV infection. Cervical HPV is a highly prevalent sexually transmitted infection in women in Spain, particularly among those under 30 years of age in whom the prevalence approaches 30%. In addition to young age, increasing number of lifetime sexual partners and a history of genital warts were identified as key risk factors for cervical HPV infection, particularly for HR HPV, reinforcing knowledge that cervical HPV infection is acquired early in a woman’s sexual life. Living in an urban community, country of birth other than Spain, low level of education, and current smoking were also identified as independent risk factors for HPV infection. A weak inverse association between condom use and HPV infection was also observed. In non-monogamous women, circumcision of the partner was associated with a reduced risk of HPV infection and smoking with an increased risk of HPV infection. Our results emphasize the importance of HPV vaccination programs for young women before they acquire sexual partners to prevent HR and LR HPV infections. Surveys of cervical HPV prevalence and its related risk factors increase our understanding of the natural history of this infection and establish the basis for future assessments of the impact of HPV immunization programs and the dynamics of risk factors for HPV infection in the population of Spain.

## Abbreviations

CI, Confidence interval; HC2, Hybrid Capture II assay; HPV, Human papillomavirus; HR, High-risk; LBC, Liquid-based cytology; LR, Low-risk; POR, Prevalence odds ratio; STI, Sexually transmitted infection.

## Competing interests

The competing interest of the authors, including financial interests and relationships and affiliations relevant to the subject of the manuscript are as follows: Esther Roura has received travel grants from GlaxoSmithKline and Sanofi Pasteur MSD. Thomas Iftner has received Institutional research grants from Gen-Probe, GlaxoSmithKline, Hologic, Roche and Sanofi Pasteur MSD. José Antonio Vidart has received travel, speaker honoraria and research grants from Sanofi Pasteur MSD. Susanne Krüger Kjaer has received lecture fees, advisory board fees and institutional research grants from Sanofi Pasteur MSD and Merck & Co. F. Xavier Bosch has received advisory board fees from Sanofi Pasteur MSD, speakers’ bureaux from GlaxoSmithKline and Sanofi Pasteur MSD and educational grants from GlaxoSmithKline and Sanofi Pasteur MSD. Nubia Muñoz has received honoraria from Merck and Sanofi Pasteur MSD as a member of Steering Committee and of Advisory board. Santiago Palacios have been a symposium speaker or advisory board member for Amgen, Arkopharma, Bayer Schering Pharma, Boehringer-Ingelheim Daiichi-Sankyo, Lilly, Pfizer, Roche, Sanofi Pasteur MSD, Servier and Warner Chilcott. Javier Cortes has received travel, speaker honoraria, and research grants from GlaxoSmithKline, Qiagen and Sanofi Pasteur MSD. Xavier Castellsagué has received travel and speaker honoraria and institutional research grants from GlaxoSmithKline, Merck & Co., Inc., and Sanofi Pasteur MSD. Maria San Martin Rodriguez, Carmen Morillo, Laurence Serradell and Laurence Torcel-Pagnon are employees of Sanofi Pasteur MSD, provider of the HPV quadrivalent vaccine approved in the European Union.

## Authors’ contributions

ER was responsible for data analysis, interpretation and writing of the manuscript. TI was responsible for trial design and data analysis, collection and interpretation. JAV was responsible for data collection and interpretation. SKK was responsible for trial design. FXB was responsible for trial design and data interpretation. NM was responsible for trial design and data interpretation. SP was responsible for data interpretation. MSMR and CM were responsible for trial design. LS was responsible for trial design and data interpretation. LTP was responsible for data interpretation, JC was responsible for data interpretation. XC was responsible for study design, data analysis, interpretation and writing of the manuscript. All authors critically reviewed the manuscript and validated the final version.

## Members of the CLEOPATRE Spain Study Group

The 77 study investigators:

Rafael Comino, Hospital Universitario de Puerto Real, Puerto Real, Spain

José Antonio Borrego, Hospital Materno Infantil Reina Sofia, Córdoba, Spain

Mónica García, Centro de Especialidades “La Bola Azul”, Almería, Spain

Rogelio Garrido, Consulta Privada, Sevilla, Spain

Francisco Montoya, Hospital Materno Infantil Virgen de las Nieves, Granada, Spain

José Antonio Vargas, Consulta Privada, Sevilla, Spain

José Luis Cuadros, Hospital Clínico Universitario San Cecilio, Granada, Spain

Blas Hervías, Hospital Universitario Puerta del Mar, Cádiz, Spain

Miguel Ochando, Complejo Hospitalario Cuidad de Jaén, Jaén, Spain

Antonio de Toro, Hospital San Juan de Dios, Bormujos, Spain

Antonino Parrilla, Centro de Especialidades Esperanza Macarena, Sevilla, Spain

Tomás Carrascal, Consulta Privada, Huelva, Spain

Pedro Abad, Hospital Provincial de Almería, Almería, Spain

José Manuel Ramón, Hospital de San Jorge, Huesca, Spain

María Isabel Morollón, Hospital Universitario Miguel Servet, Zaragoza, Spain

Luis González, Consulta Privada, Oviedo, Spain

Concepción Solares, Centro de Salud del Quirinal, Avilés, Spain

Javier Barrés, Policlínica Miramar, Palma de Mallorca, Spain

Gabriel Ferret, Clínica Miramar, Palma de Mallorca, Spain

Orlando Falcón, Instituto Falcón Vizcaíno, Las Palmas, Spain

Lucía Almeida, Hospital Ntra Sra. De la Candelaria, Santa Cruz de Tenerife, Spain

Juan Miguel Falcón, Clínica de obstetricia y ginecología Dr. Falcón, Telde, Spain

José Bretones, Residencia Hospital Marqués de Valdecilla, Santander, Spain

José Antonio Mínguez, Consulta Privada, Valladolid, Spain

Guadalupe Bombín, Hospital Río Carrión, Palencia, Spain

Jaime Moreno, Hospital Vírgen de la Concha, Zamora, Spain

Diana Fernández, Hospital General de Segovia, Segovia, Spain

Gaspar González, Complejo Hospitalario Universitario de Albacete, Albacete, Spain

Carlos Zorzo, Hospital Gral. De Guadalajara, Guadalajara, Spain

Manuel Sánchez, Consulta Privada, Toledo, Spain

Montserrat Cararach, USP Institut Universitari Dexeus, Barcelona, Spain

Jordi Antoni, Hospital Teknogin, Barcelona, Spain

Pere Cavallé, Hospital Universitario Sant Joan de Reus, Tarragona, Spain

Pere Fusté, A.G. GINOCOLEGS, Barcelona, Spain

Jordi Xercavins, Hospital Universitari Vall d’Hebrón, Barcelona, Spain

Cristina Centeno, Hospital Universitari Vall d’Hebrón, Barcelona, Spain

María Eulalia Fernández, Centro de Atención Primaria Ramona Vía, El Prat de Llobregat, Spain

Aureli Torné, Hospital Clinic i Provincial, Barcelona, Spain

Luis Puig, Consulta Privada, Barcelona, Spain

Ramón Carreras, Hospital Mare de Deu del Mar, Barcelona, Spain

Joan Meléndez, Meléndez Ginecológica SC, Blanes, Spain

Juan Carlos Martínez, Centro de Salud S. Vicente del Raspeig, Alicante, Spain

Ana de Gonzalo, Centro de Especialidades Monteolivete, Valencia, Spain

Ana Boldo, Hospital La Plana, Villareal, Spain

Alberto Romeu, Centro de Salud de Godella, Godella, Spain

José Antonio López, Consulta Ginecológica, Alicante, Spain

Mar Sanz, Centro de Planificación Familiar “El Carmen”, Manises, Spain

José María Galán, Hospital Gral. San Pedro de Alcántara, Cáceres, Spain

Francisco Solano, Hospital Materno-Infantil Perpetuo Socorro, Badajoz, Spain

Alejandro Novo, H. C. U. de Santiago de Compostela, Santiago de Compostela, Spain

José Luis Gómez, H. Materno-Infantil Teresa Herrera, La Coruña, Spain

Concepción Moreno, Centro de Orientación Familiar del Ferrol, Ferrol, Spain

Angel de la Orden, Hospital Do Meixoeiro, Vigo, Spain

Francisco Vázquez, Clínica Ginecológica CEOGA, Lugo, Spain

Pilar Miranda, Hospital de Fuenlabrada, Fuenlabrada, Spain

Pilar Benavides, Hospital Universitario Santa Cristina, Madrid, Spain

Emilia de Dios, Centro de Salud Dos de Mayo, Móstoles, Spain

José Manuel Hernández, Hospital 12 de Octubre, Madrid, Spain

José Miguel Seoane, Hospital 12 de Octubre, Madrid, Spain

Miguel Angel Huertas, Hospital Universitario de Getafe, Getafe, Spain

Mónica García, Fundación Hospital de Alcorcón, Alcorcón, Spain

Juan Ordás, Centro de Especialidades José Marvá, Madrid, Spain

Dolores Rubio, Hospital Ramón y Cajal, Madrid, Spain

Esperanza Díaz, Hospital Ramón y Cajal, Madrid, Spain

Juan Carlos Recio, Centro de Salud Andrés Mellado, Madrid, Spain

Álvaro Zapico, OBS-GYN S.L., Murcia, Spain

José Ramón Rodriguez, Consulta Privada, Murcia, Spain

José Ramón Rodriguez, Hospital Los Arcos, Santiago de la Ribera, Spain

Juan Carlos Muruzábal, Instituto Navarro de Ginecología, Pamplona, Spain

Daniel Andía, Hospital de Basurto, Bilbao, Spain

Enrique Izaguirre, Hospital Txagorritxu, Vitoria, Spain

Borja Rivero, Consulta Privada, Zarauz, Spain

José Ramón Serrano, Clínica San Martín Osasunaegía, Berbara, Spain

Esteban Campeny, Consulta Privada, Logroño, Spain

## Pre-publication history

The pre-publication history for this paper can be accessed here:

http://www.biomedcentral.com/1471-2334/12/145/prepub
